# *FAM83A* is amplified and promotes cancer stem cell-like traits and chemoresistance in pancreatic cancer

**DOI:** 10.1038/oncsis.2017.3

**Published:** 2017-03-13

**Authors:** S Chen, J Huang, Z Liu, Q Liang, N Zhang, Y Jin

**Affiliations:** 1 Department of Hepatobiliary Surgery, The Third Affiliated Hospital of Sun Yat-Sen University, Guangzhou, China; 2 Department of Gastrointestinal Surgery, The Third Affiliated Hospital of Sun Yat-Sen University, Guangzhou, China; 3 Department of Emergency Medicine, The Third Affiliated Hospital of Sun Yat-Sen University, Guangzhou, China; 4 Department of Pathology, The Third Affiliated Hospital of Sun Yat-Sen University, Guangzhou, China

## Abstract

Cancer stem cells (CSCs), also known as tumor-initiating cells (TICs), contribute to tumorigenesis, resistance to chemoradiotherapy and recurrence in human cancers, suggesting targeting CSCs may represent a potential therapeutic strategy. In the current study, we found family with sequence similarity 83, member A (FAM83A) is significantly overexpressed and associated with poorer overall survival and disease-free survival in pancreatic cancer. Overexpression of FAM83A markedly promoted, whereas inhibition of FAM83A decreased, CSC-like traits and chemoresistance both *in vitro* and in an *in vivo* mouse model of pancreatic cancer. Furthermore, overexpression of FAM83A activated the well-characterized CSC-associated pathways transforming growth factor-β (TGF-β) signaling and Wnt/β-catenin signaling. Importantly, the *FAM83A* locus was amplified in a number of human cancers and silencing *FAM83A* in associated cancer cell lines inhibited activation of the WNT/β-catenin and TGF-β signaling pathways and reduced tumorigenicity. Taken together, these results indicate that FAM83A has a vital oncogenic role to promote pancreatic cancer progression and may represent a potential clinical target.

## Introduction

Pancreatic cancer is the seventh leading cause of cancer-related mortality.^1, 2^ Despite advances in modern medical technology, pancreatic cancer has benefited from marginal improvements in survival outcomes; the 5-year overall survival rate of patients with pancreatic cancer is only 6% and the median survival time is <9 months.^3, 4^ Failure of conventional chemotherapy, including both intrinsic and acquired chemoresistant behavior, is a major factor that significantly decreases the clinical efficacy of chemotherapy for pancreatic cancer.^5, 6^ The response rates to common chemotherapeutic drugs, such as gemcitabine, erlotinib and 5-fluorouracil (5-FU), in pancreatic cancer have been reported to be lower than 25%.^5, 7, 8^ Therefore, better understanding the molecular mechanisms that underlie drug resistance in pancreatic cancer could lead to the development novel therapeutic strategies for this highly lethal malignancy.

The intrinsic resistance of cancer stem cells (CSCs), also known as tumor-initiating cells (TICs), to conventional therapy is currently regarded as a potential therapeutic target.^9^ For instance, it has recently been reported that the high rates and patterns of therapeutic failure observed in ovarian cancer are closely associated with stable accumulation of drug-resistant CSCs.^10^ Li *et al.*^11^ found that the percentage of the CD44^+^CD24^–/low^ CSC sub-population, which exhibits intrinsic resistance to chemotherapy, was significantly increased in patients with breast cancer treated with chemotherapeutic drugs such as docetaxel, doxorubicin or cyclophosphamide. Similarly, CD133^+^ pancreatic CSCs have been demonstrated to be exclusively tumorigenic and highly resistant to chemotherapy and radiation therapy, and the CD133^+^ CXCR4^+^ sub-population of pancreatic CSCs is critical for tumor metastasis,^12, 13, 14^ suggesting that CSCs have important roles in pancreatic cancer progression. Therefore, targeting pancreatic CSCs could potentially increase chemosensitivity and thus improve the response to treatment.

Family with sequence similarity 83, member A (*FAM83A*), also known as *BJ-TSA-9*, is located on chromosome 8q24 and was originally identified as a potential tumor-specific gene by a bioinformatics approach.^15^ Furthermore, FAM83A is overexpressed in multiple human tumors, including lung, breast, testis and bladder cancer,^16, 17, 18^ suggesting that FAM83A may have an oncogenic role during the development and progression of cancer. Moreover, using a 3D phenotypic reversion assay, Lee *et al.*^19^ identified that FAM83A may contribute to resistance to tyrosine kinase inhibitors in breast cancer through activation of the epidermal growth factor receptor/phosphatidylinositol 3 kinase/AKT signaling pathway via interacting with c-RAF and phosphatidylinositol 3 kinase p85,^20^ indicating that overexpression of FAM83A may lead to chemoresistance. Concordantly, silencing *FAM83A* markedly decreased the proliferation, anchorage-independent growth and invasion capabilities of breast cancer cells both *in vitro* and *in vivo*,^19^ further supporting the suggestion that FAM83A represents a potential target for cancer therapy.

Herein, we report that FAM83A is markedly overexpressed in pancreatic cancer cell lines and clinical tissues. Importantly, silencing *FAM83A* markedly decreased pancreatic CSC-like traits *in vitro* and tumorigenicity *in vivo* via inhibition of two well-established CSC-associated signaling pathways, transforming growth factor-β (TGF-β) and Wnt/β-catenin. Therefore, this study indicates FAM83A exerts a critical oncogenic role in pancreatic cancer progression and may represent a potential clinical target for cancer therapy.

## Results

### Overexpression of FAM83A in pancreatic cancer is associated with poor prognosis

**Figure 1:**
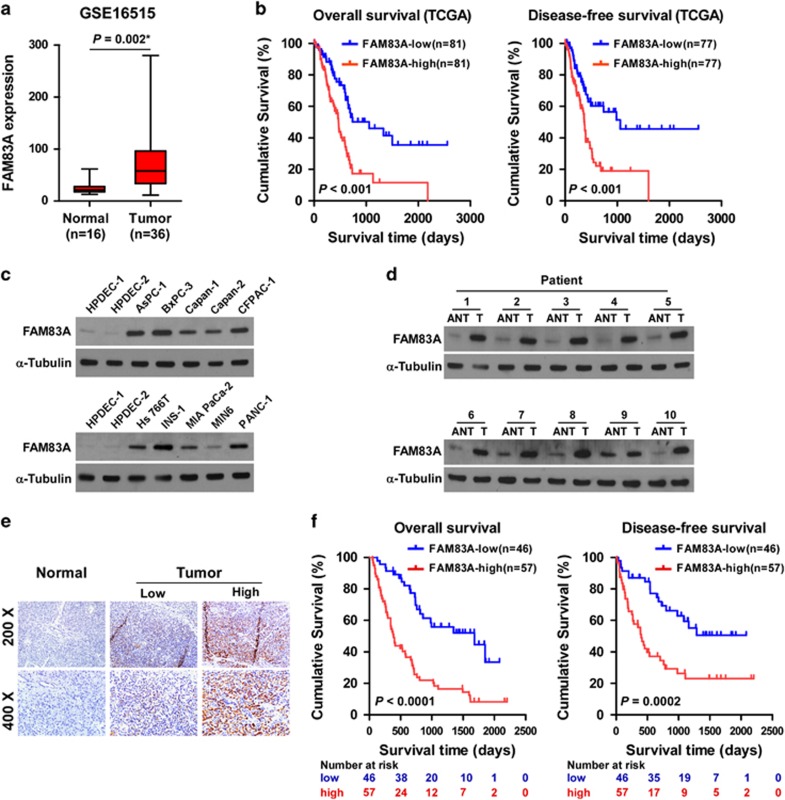
FAM83A is overexpressed in pancreatic cancer cell lines and primary human pancreatic cancer. (**a**) *FAM83A* mRNA expression in a published microarray data set (NCBI/GEO/GSE16515; contains 16 normal and 36 pancreatic tumor samples). (**b**) Kaplan–Meier analysis of overall (left) or disease-free (right) survival for patients with pancreatic cancer in the TCGA data set with low vs high *FAM83A* expression; *P<0.05. (**c**, **d**) Western blotting analysis of FAM83A expression in 2 primary normal human pancreatic duct epithelial cell (HPDEC) lines and 10 cultured pancreatic cancer cell lines (**c**) and 10 primary pancreatic cancer tissues (T) and the matched adjacent non-tumor tissues (ANT) (**d**); α-tubulin was used as protein loading control. (**e**) Immunohistochemical staining showing FAM83A protein expression was upregulated in human pancreatic cancer specimens compared with normal pancreatic tissues. (**f**) Kaplan–Meier overall (left) and disease-free (right) survival curves for patients with pancreatic cancer with low vs high FAM83A expression (*n*=103; *P*<0.001, log-rank test).

By analyzing a published microarray data set (NCBI/GEO/GSE16515; *n*=52, containing 16 non-tumor and 36 tumor samples), we found that *FAM83A* messenger RNA (mRNA) was significantly upregulated in pancreatic cancer tissues compared with normal pancreatic tissues (Figure 1a). Furthermore, analysis of The Cancer Genome Atlas (TCGA) data sets revealed patients with higher *FAM83A* expression had poorer overall survival and disease-free survival (*P*<0.001, *P*<0.001; Figure 1b), suggesting that FAM83A may have a critical role in pancreatic cancer progression.

In agreement, FAM83A was upregulated at both the protein and mRNA levels in all ten pancreatic cancer cell lines analyzed compared with 2 primary normal human pancreatic duct epithelial cell lines, and in 10 human pancreatic cancer samples compared with the matched adjacent non-tumor tissues (Figures 1c and d and Supplementary Figures S1a and b). Immunohistochemical analysis confirmed FAM83A was markedly upregulated in pancreatic cancer tissues (*n*=103) but barely detectable in normal pancreatic tissues (*n*=10; Figure 1e), and FAM83A protein expression was positively associated with clinical stage (*P*=0.005), tumor-node-metastasis (TNM) classification (T: *P*=0.010; N: *P*=0.004; M: *P*=0.013) and histological differentiation (*P*=0.014) in pancreatic cancer (Supplementary Table S2). Furthermore, Kaplan–Meir analysis and the log-rank test demonstrated that patients with pancreatic cancer with high FAM83A expression had significantly poorer overall and disease-free survival than patients with low FAM83A expression (Figure 1f; *P*<0.001; *P*=0.0002). Univariate and multivariate analyses revealed FAM83A expression was an independent prognostic factor for overall survival (Supplementary Table S3; univariate analysis: hazard ratio, 2.836; 95% confidence interval, 1.876–4.286, *P*<0.001; multivariate analyses: hazard ratio, 2.212; 95% confidence interval, 1.420–3.411, *P*=0.002), and an independent prognostic factor for disease-free survival (Supplementary Table S4; univariate analysis: hazard ratio, 3.267; 95% confidence interval, 2.165–5.145, *P*<0.001; multivariate analyses: hazard ratio, 2.412; 95% confidence interval, 1.582–3.874, *P*=0.01), in pancreatic cancer. Taken together, these results indicate that FAM83A exerts an oncogenic role during pancreatic cancer progression.

### Upregulation of FAM83A promotes pancreatic CSC-like traits *in vitro*

**Figure 2:**
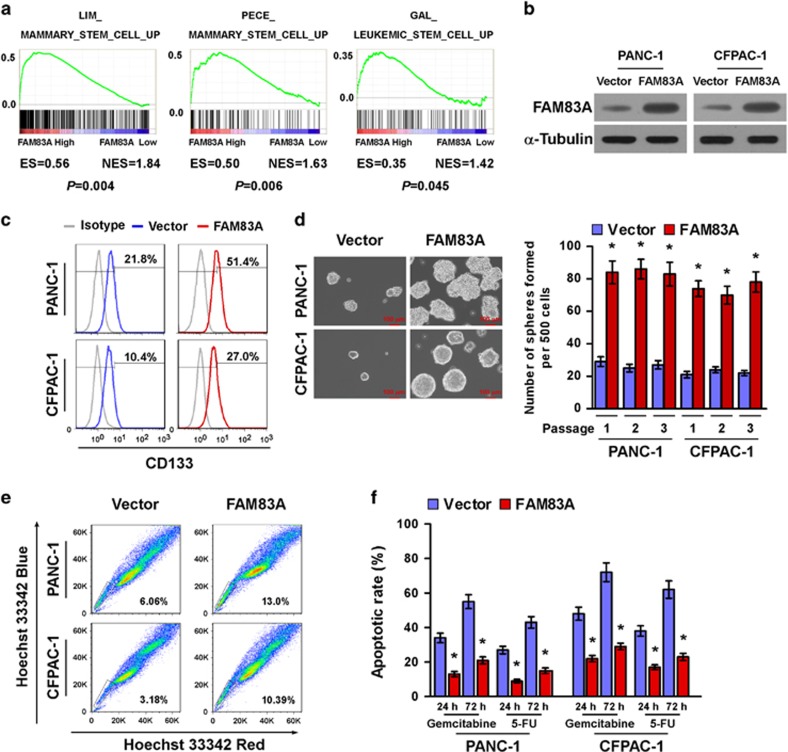
Upregulation of FAM83A promotes pancreatic CSC-like traits *in vitro.* (**a**) Gene set enrichment analysis (GSEA) plot showing positive correlations between high *FAM83A* expression and stem cell gene signatures (LIM_MAMMARY_STEM_CELL_UP; GAL_LEUKEMIC_STEM_CELL_UP; PECE_ MAMMARY_STEM_CELL_UP) in a published pancreatic cancer data set (GSE16515). (**b**) Western blotting analysis of FAM83A expression in PANC-1 and CFPAC-1 pancreatic adenocarcinoma cells stably expressing FAM83A cDNA; α-tubulin was used as loading control. (**c**) Flow cytometry analysis of the CD133^+^ population in the indicated cells. (**d**) Representative images of tumorspheres formed by the indicated cells (left); scale bar: 100 μm. Histograms (right) showing the mean number of spheres formed. (**e**) Hoechst 33342 dye exclusion assay showing FAM83A overexpression increased the number of SP^+^ cells. (**f**) Effects of chemotherapeutic drugs on the indicated pancreatic cancer cells. Pancreatic cancer cells were seeded in culture plates and incubated with and without 50 μM gemcitabine and 5-FU for 24 or 72 h. Apoptotic cells were measured by FACS analysis. Each bar is the mean±s.d. of three independent experiments; *P<0.05.

Gene set enrichment analysis of the GSE16515 data set revealed a remarkable overlap between profiles with high *FAM83A* expression and stem cell gene signatures (Figure 2a), suggesting FAM83A may be involved in the regulation of CSC-like traits. In agreement with this hypothesis, overexpressing FAM83A in pancreatic cancer cells markedly increased the CD133^+^ population (Figures 2b and c), which is exclusively tumorigenic and highly chemoresistant.^12^ Moreover, FAM83A-transduced cells formed significantly larger and higher numbers of spheres in the tumorsphere formation assay compared with vector control cells (Figure 2d). In addition, overexpression of FAM83A significantly upregulated the mRNA expression levels of multiple pluripotency factors, including *ABCG2*, *BMI1*, *SOX2*, *OCT4* and *NANOG* (Supplementary Figure S2a). Furthermore, overexpressing FAM83A in pancreatic cancer cell lines significantly increased the proportions of SP^+^ cells, a sub-population of cells that can exhibit drug resistance and have CSC-like characteristics (Figure 2e).^21^ In agreement with this observation, FAM83A-transduced cells exhibited higher resistance to chemotherapeutic drugs such as gemcitabine and 5-FU (Figure 2f). Taken together, these results show FAM83A promotes a CSC-like phenotype and enhances chemoresistance in pancreatic cancer cells *in vitro*.

### Silencing FAM83A inhibits pancreatic CSC-like traits *in vitro*

**Figure 3:**
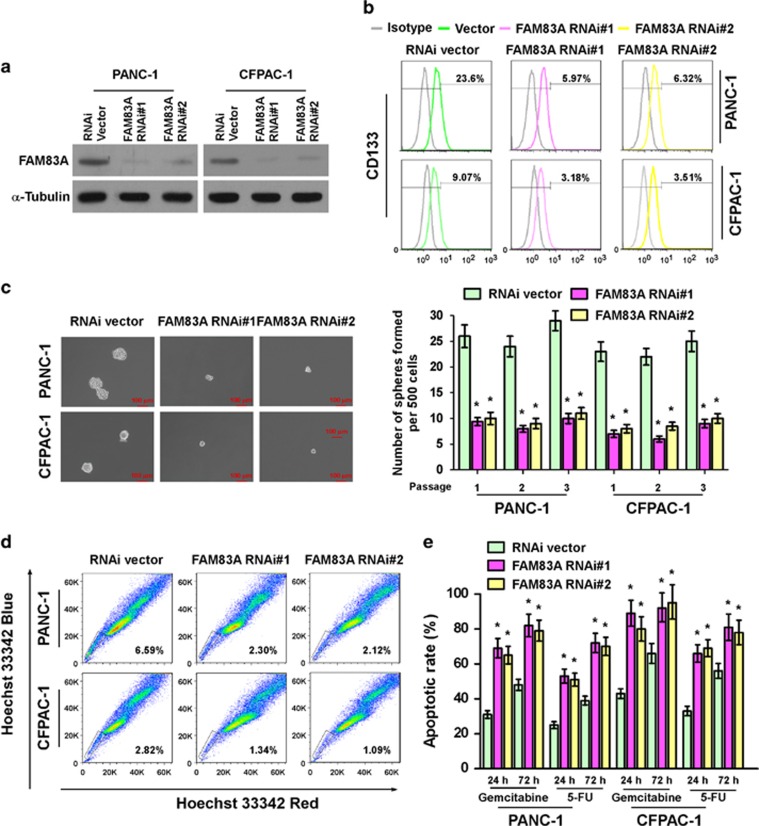
Silencing *FAM83A* inhibits pancreatic CSC-like traits *in vitro*. (**a**) Western blotting analysis of FAM83A expression in PANC-1 and CFPAC-1 pancreatic cancer cells stably expressing *FAM83A* shRNAs. (**b**) Flow cytometry analysis of the CD133^+^ population in the indicated cells. (**c**) Representative images of the tumorspheres formed by the indicated cells (left). Scale bar: 100 μm. Histograms (right) showing the mean number of spheres formed. (**d**) Hoechst 33342 dye exclusion assay showing that silencing *FAM83A* decreased the number of SP^+^ cells. (**e**) Effects of chemotherapeutic drugs on pancreatic cancer cells. Pancreatic cancer cells were seeded in culture plates and incubated with and without 50 μM gemcitabine and 5-FU for 24 and 72 h. Apoptotic cells were measured by FACS analysis. Each bar represents the mean±s.d. of three independent experiments; *P<0.05.

In agreement with the gain-of-function experiments, silencing *FAM83A* in pancreatic cancer cell lines significantly decreased the proportion of CD133^+^ cells, tumorsphere number and size, the proportion of SP^+^ cells, resistance to gemcitabine and 5-FU (Figures 3a–e) and the mRNA expression levels of pluripotency factors compared with RNAi-vector control cells (Supplementary Figure S2b). Collectively, these results further support the notion that FAM83A has an important role in maintenance of the CSC-like phenotype in pancreatic cancer *in vitro*.

### FAM83A promotes pancreatic cancer tumorigenesis and chemoresistance *in vivo*

**Figure 4:**
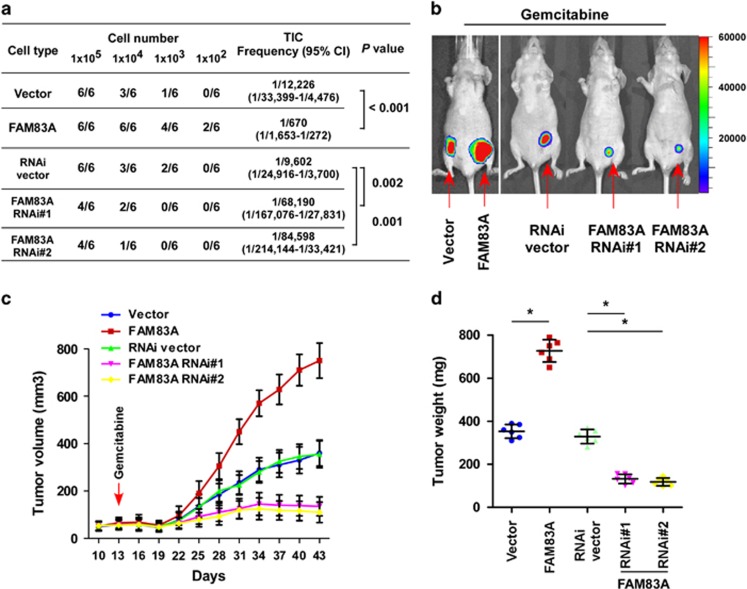
FAM83A promotes pancreatic cancer tumorigenesis and chemoresistance *in vivo.* (**a**) Tumor formation rate for different dilutions and estimated percentages of CSCs. (**b**) Representative images of tumor-bearing mice in each group. (**c**) Representative tumor growth curves of xenografts derived from each group treated with gemcitabine (80 mg/kg). (**d**) Tumor size; results are mean±s.d. of three independent experiments; *P<0.05.

The effect of FAM83A on pancreatic CSC-like traits was further examined using an *in vivo* tumor model. As shown in Figure 4a, extreme limiting dilution analysis revealed FAM83A-transduced cells formed a significantly higher incidence of tumors and had a greater tumorigenic capability; PANC-1/FAM83A cells formed tumors even when only 1 × 10^2^ cells were implanted. In contrast, FAM83A RNAi cells had a significantly lower tumor-initiating capacity (Figure 4a).

The effect of FAM83A on chemoresistance was also examined *in vivo*. As shown in Figures 4b and d, the tumors formed by PANC-1/FAM83A cells had significantly higher volumes and weights after the tumor-bearing mice were treated intraperitoneally with gemcitabine (compared with tumors formed by control cells; *P*<0.05). Conversely, depletion of endogenous *FAM83A* in PANC-1 cells significantly enhanced the sensitivity of the resulting tumors to gemcitabine, as indicated by both tumor volume and weight (both *P*<0.05). These results further show FAM83A has important roles in pancreatic cancer tumorigenesis and chemoresistance.

### Overexpression of FAM83A activates the Wnt/β-catenin and TGF-β signaling pathways

**Figure 5:**
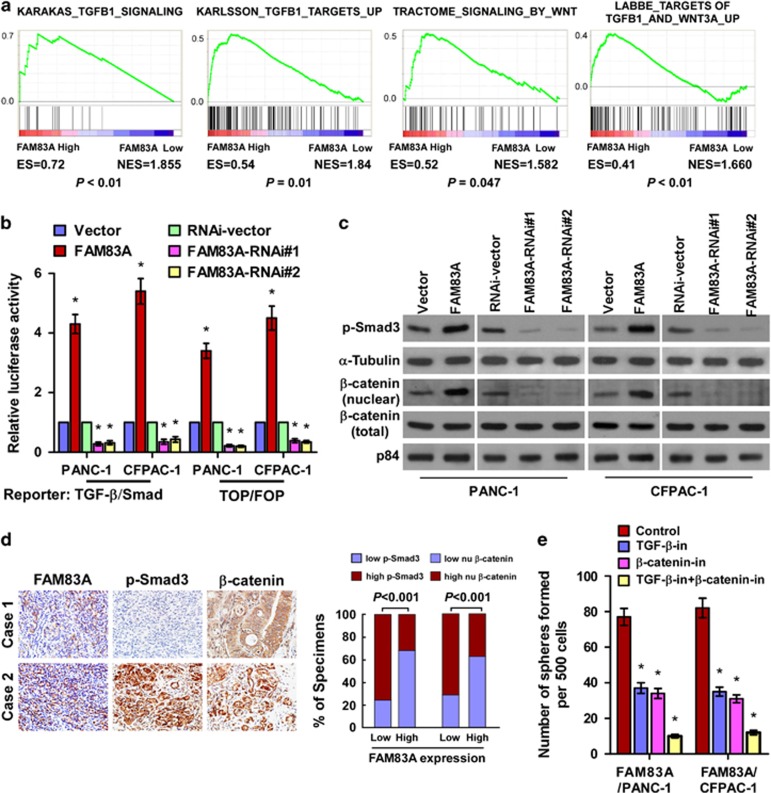
Overexpression of FAM83A activates multiple CSC-associated signaling pathways. (**a**) Gene set enrichment analysis (GSEA) plot showing the positive correlations between high *FAM83A* expression and TGF-β/Smad and Wnt/β-catenin pathway gene signatures (KARAKAS_TGFB1_SIGNALING; KARLSSON_TGFB_TARGETS_UP; TRACTOME_SIGNALING_BY WNT; LABBE_TARGETSOF TGFB1_AND_WNT3A_UP) in published TCGA data sets (*n*=178). (**b**) Relative luciferase activity of TGF-β reporter and TOP/FOP reporter genes. (**c**) Western blotting (WB) analysis of p-Smad3 (Ser423/425) and nuclear β-catenin in the indicated cells; α-tubulin was used as the loading control. (**d**) WB of p-Smad3 and nuclear β-catenin expression in ten freshly isolated pancreatic cancer tissues; α-tubulin was used as the loading control. (**e**) Histograms (right) of the mean number of spheres formed by FAM83A-transduced cells treated with a TGF-β inhibitor and/or β-catenin inhibitor. Each bar represents the mean±s.d. of three independent experiments; *P<0.05.

To explore the mechanism underlying the ability of FAM83A to promote pancreatic CSC-like traits, gene set enrichment analysis was performed using the TCGA data set for pancreatic cancer. *FAM83A* expression correlated significantly with activated gene signatures for both the Wnt/β-catenin and TGF-β/Smad pathways (Figure 5a), suggesting these well-characterized CSC-associated signaling pathways^9, 22, 23^ function downstream of FAM83A to promote pancreatic CSC-like traits.

In agreement with this suggestion, overexpressing FAM83A significantly enhanced, whereas silencing *FAM83A* significantly reduced, the activities of β-catenin-driven and TGF-β-driven luciferase reporter genes and the expression of numerous downstream genes in both pathways (Figure 5b and Supplementary Figure S3a). In addition, the expression of nuclear β-catenin and phosphorylated-Smad3 (p-Smad3, (er423/425) were markedly elevated in FAM83A-transduced cells and downregulated in *FAM83A*-silenced cells (Figure 5c). Furthermore, the expression of FAM83A was strongly associated with the levels of p-Smad3 (*P*<0.001) and nuclear β-catenin (*P*<0.001; Figure 5d), indicators of TGF-β/Smad and Wnt/β-catenin pathway activation, respectively, in 103 paraffin-embedded pancreatic adenocarcinoma samples. Moreover, the levels of p-Smad3 and nuclear β-catenin were inversely associated with overall survival in pancreatic cancer (Supplementary Figure S3b). Consistent with these data, FAM83A levels correlated positively with nuclear β-catenin (*r*=0.63; *P*<0.05) and p-Smad3 expression (*r*=0.77; *P*<0.05) in 10 freshly collected clinical pancreatic cancer samples, further suggesting FAM83A expression is clinically associated with Wnt/β-catenin and TGF-β/Smad pathway activation in pancreatic cancer (Supplementary Figure S3c). Importantly, inhibiting the Wnt/β-catenin pathway using a β-catenin inhibitor or TGF-β/Smad signaling with a TGF-β inhibitor significantly decreased the tumorsphere formation capability of FAM83A-transduced cells (Figure 5e), confirming the Wnt/β-catenin and TGF-β/Smad pathways are functional effectors of FAM83A-induced CSC-like traits.

### FAM83A is amplified in pancreatic cancer and a subset of cancers

**Figure 6:**
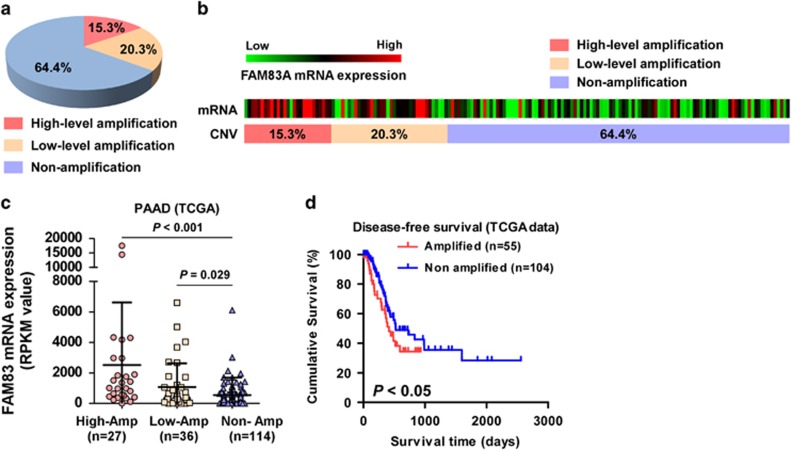
Aberrant FAM83A contributes to progression of various types of cancer. (**a**) Analysis of *FAM83A* CNV in TCGA data sets revealed the *FAM83A* locus is amplified in 35.5% of pancreatic cancer samples. (**b**) *FAM83A* gene CNV and corresponding mRNA expression in a TCGA pancreatic cancer data set (*P*<0.05, *n*=177). (**c**) *FAM83A* gene CNV and corresponding mRNA expression in a TCGA pancreatic cancer data set (*P*<0.05, *n*=177). (**d**) Kaplan–Meier analysis of overall or disease-free survival for patients with low or high FAM83A expression.

The *FAM83A* locus is located on chromosome 8q24.13, which is frequently amplified in a number of human cancers.^24, 25, 26, 27^ Analysis of *FAM83A* copy number variation (CNV) in the TCGA data set revealed the *FAM83A* locus was amplified in 37.3% of pancreatic cancer samples and *FAM83A* mRNA expression was significantly associated with *FAM83A* CNV (Figures 6a and c). Furthermore, patients with pancreatic cancer who had *FAM83A* amplification had poorer survival outcomes than patients without *FAM83A* amplification (*P*=0.043; Figure 6d).

Interestingly, analysis of the TCGA data sets revealed *FAM83A* CNV and *FAM83A* mRNA levels were also markedly increased in subtypes of a number of primary tumor types, including lung adenocarcinoma and cervical, bladder, breast, colon, head and neck, pancreatic and uterine cancer (Supplementary Figures S4a and b). Moreover, the levels of *FAM83A* mRNA were significantly upregulated and a positive correlation was observed between *FAM83A* mRNA levels and *FAM83A* CNV in lung adenocarcinoma and cervical cancer (Supplementary Figure S4c). In agreement with the observations in pancreatic cancer, higher *FAM83A* expression was associated with poorer overall survival in lung adenocarcinoma and cervical and uterine cancer (Supplementary Figure S4d). These results suggest overexpression of FAM83A contributes to progression in several types of cancer.

The oncogenic function of FAM83A in other human cancers was further examined in lung adenocarcinoma and cervical squamous cell carcinoma and endocervical adenocarcinoma (CESC), the cancers in which *FAM83A* was most frequently amplified. As shown in Figure 6f, overexpressing FAM83A significantly increased, whereas silencing *FAM83A* decreased, β-catenin-driven and TGF-β-driven luciferase reporter activity in both lung adenocarcinoma and CESC cell lines (Supplementary Figure S4f). Moreover, FAM83A-transduced lung adenocarcinoma and CESC cells formed significantly larger and higher numbers of tumors in the tumorsphere formation assay, whereas *FAM83A*-silenced cells formed smaller and lower numbers of tumorspheres, compared with the respective control cells (Supplementary Figure S4g). Taken together, these results show aberrant amplification of *FAM83A* promotes CSC-like traits by activating the Wnt/β-catenin and TGF-β pathways, resulting in poorer clinical outcomes in patients with cancer.

## Discussion

The findings of the present study provide new insight into a potential oncogenic role for FAM83A in pancreatic cancer progression and highlight the ability of FAM83A to promote pancreatic CSC-like traits. We demonstrate FAM83A is significantly overexpressed in pancreatic cancer and promotes CSC-like traits by activating the Wnt/β-catenin and TGF-β pathways. The mechanism of FAM83A upregulation in pancreatic cancer can be attributed to amplification of the genomic locus on chromosome 8q24.13. Hence, our results uncover a novel mechanism leading to overexpression of FAM83A in pancreatic cancer, and suggest this protein has potential as a therapeutic target for pancreatic cancer.

The contribution of CSCs to tumor metastasis, recurrence and chemoradiotherapeutic resistance suggests targeting this sub-population of tumor cells could be an effective strategy to improve the survival outcomes of patients with lethal malignancies.^28, 29^ Identifying key targets to eliminate CSCs or inhibit CSC traits could attenuate tumor cell resistance to conventional cytotoxic therapies, and reduce metastasis and relapse. For instance, Wang *et al.*^30^ reported that eliminating CSCs in hematological malignancies using a hypoxia-inducible factor-α inhibitor could eradicate mouse lymphoma and serially transplantable human acute myeloid leukemia in xenogeneic models; this strategy could provide an effective approach for treating hematological malignancies. Sachlos *et al.*^31^ reported that selectively targeting leukemia stem cells treated with a dopamine receptor antagonist induced neoplastic human pluripotent stem cell differentiation, impaired the ability of human somatic CSCs to initiate leukemia *in vivo* and augmented the effects of chemotherapeutic drugs, suggesting that inducing CSC differentiation could also represent a strategy for treating cancer.^31^ Jin *et al.*^32^ demonstrated that a CD44 monoclonal antibody targeting CD44, a key regulator of leukemic stem cells, eliminated quiescent acute myeloid leukemia and leukemic stem cells and markedly reduced leukemic repopulation. Therefore, identification of the key regulatory effector(s) of CSC traits may provide potential target(s) for cancer treatment. Herein, we found that silencing *FAM83A* markedly decreased CSC-like traits and tumorigenicity and significantly enhanced the sensitivity of pancreatic cancer cells to chemotherapeutic drugs. These results not only provide mechanistic insight into the regulation of CSC-like properties in pancreatic cancer, but may also provide a novel target for pancreatic cancer treatment.

Although FAM83A has previously been reported to have an important role in therapeutic resistance to tyrosine kinase inhibitors in breast cancer, numerous studies have demonstrated oncogenic *K-RAS* mutations are the major mechanism of epidermal growth factor receptor-mediated resistance in pancreatic cancer;^33, 34^ approximately 90% of patients with pancreatic cancer harbor gain-of-function *K-RAS* mutation.^35^ Therefore, FAM83A-induced pancreatic progression may be mediated via other mechanisms. Numerous recent studies have demonstrated multiple signaling pathways, such as the TGF-β and Wnt/β-catenin pathways, are required for self-renewal and maintenance of the CSC phenotype.^36, 37, 38^ Activation of the TGF-β pathway has been reported to contribute to tumor heterogeneity and chemoresistance in squamous cell carcinoma CSCs, leading to tumor recurrence.^23^ Inhibition of the TGF-β pathway decreases the CD44^high^/Id1^high^ glioma-initiating cell population and reduces the capacity of glioma-initiating cells to initiate tumors,^39, 40^ suggesting TGF-β is a key regulator of CSCs. Moreover, Wnt/β-catenin signaling also has an important role in the regulation of CSC self-renewal and tumorigenesis.^41^ Excessive Wnt/β-catenin signaling is required to maintain CSC capabilities in colon cancer, glioma and mixed lineage leukemia.^22^ Importantly, Scheel *et al.*^42^ reported TGF-β and Wnt signaling interact to induce activation of the epithelial–mesenchymal transition and maintain a CSC-like state, and inhibition of both the TGF-β and Wnt/β-catenin pathways significantly inhibited the metastatic and self-renewal abilities of primary mammary epithelial cells. Therefore, inhibition of a single CSC-associated pathway may not be sufficient to abrogate the CSC population within a tumor. Herein, we found that overexpressing FAM83A activated both the TGF-β and Wnt/β-catenin pathways and promoted CSC-like straits in pancreatic cancer, suggesting FAM83A maintains CSC properties by simultaneously activating multiple CSC-associated pathways. The precise mechanisms by which FAM83A activates the TGF-β and Wnt/β-catenin pathways are currently being investigated in our laboratory.

This study demonstrates the elevated FAM83A expression observed in numerous tumor types could be due to genomic amplification of 8q24, which is known to contain the oncogene Myc. Interestingly, TCGA analysis revealed up to 90.28% of the pancreatic cancer samples with *FAM83A* gene amplification also had *c-MYC* gene amplification. Furthermore, overexpressing c-MYC significantly increased the tumorsphere formation ability of FAM83A-transduced cells, whereas silencing *c-MYC* only partially abrogated FAM83A-induced tumorsphere formation (data not shown), suggesting FAM83A and c-MYC cooperate to promote pancreatic cancer progression via different mechanisms. Therefore, understanding the exact role of FAM83A in the pathogenesis of pancreatic cancer and the molecular mechanisms by which FAM83A activates the TGF-β and Wnt signaling pathways will increase our knowledge of the biological basis of cancer progression and may enable the development of new therapeutic strategies for patients with this lethal disease.

## Materials and methods

### Cells

Primary cultures of normal human pancreatic duct epithelial cells were established from fresh specimens of the adjacent non-tumor pancreatic tissue and maintained in bronchial epithelial basal medium (Lonza Walkersville, Walkersville, MD, USA) containing 10% fetal bovine serum and supplemented with BEGM Single-Quots (Lonza Walkersville), according to previous reports.^43, 44^ The human pancreatic cell lines AsPC-1, BxPC-3, Capan-1, Capan-2, CFPAC-1, Hs 766T, INS-1, MIA PaCa-2, MIN6 and PANC-1 were cultured as American Type Culture Collection (ATCC, Manassas, VA, USA) protocol described. All cell lines were authenticated by short tandem repeat fingerprinting at Medicine Lab of Forensic Medicine Department of Sun Yat-Sen University (Guangzhou, China) and tested for mycoplasma contamination.

### Patient information and tissue specimens

A total of 103 paraffin-embedded pancreatic adenocarcinoma samples, which were histopathologically and clinically diagnosed, were obtained from the third Affiliated Hospital of Sun Yat-Sen University. Prior patient consent and approval from the Institutional Research Ethics Committee were obtained before research purposes. Clinical information on the samples is summarized in Supplementary Table S1. Ten pancreatic adenocarcinoma specimens and the matched adjacent non-cancerous pancreatic tissues were frozen and stored in liquid nitrogen until further use. Prior patient consent and approval from the Institute Research Ethics Committee were obtained for the use of clinical materials for research purposes.

### Western blotting analysis

Western blotting was performed using anti- FAM83A antibody (Sigma, Saint Louis, MO, USA; SAB1103067), anti-p-Smad3 (Ser423/425) (Cell Signaling Technology, Danvers, MA, USA; #9520), total Smad3 (Cell Signaling Technology; #9513) and anti-β-catenin (Cell Signaling Technology; #9562) antibodies. The blotting membranes were stripped and re-probed with an anti-α-tubulin antibody or anti-p84 as a protein loading control (Sigma; SAB2701984).

### DNA/RNA extraction, reverse transcription and real-time PCR

The genomic DNA in cultured cells and pancreatic adenocarcinoma tissue samples was extracted using QIAamp DNA Mini Kit (Qiagen, Valencia, CA, USA; cat.51306) according to the manufacturer’s instructions. Total RNA from cultured cells and pancreatic adenocarcinoma tissue samples was extracted using TRIzol (Life Technologies, Grand Island, NY, USA) according to the manufacturer’s instructions. mRNA was polyadenylated using a poly-A polymerase-based First-Strand Synthesis kit (TaKaRa, DaLian, China) and reverse transcription of total mRNA was performed using a PrimeScript RT Reagent kit (TaKaRa) according to the manufacturer’s protocol. Complementary DNAs/DNAs were amplified and quantified in Bio-Rad CFX qRT–PCR detection system (Applied Biosystems Inc., Foster City, CA, USA), using FastStart Universal SYBR Green Master (ROX; Roche, Toronto, ON, Canada). Expression data were normalized to the geometric mean of housekeeping gene *GAPDH* to control the variability in expression levels and calculated as 2–[(C_t_ of gene)–(C_t_ of GAPDH)], where C_t_ represents the threshold cycle for each transcript.

### Primers and oligonucleotides

Cloning primer human FAM83A-ORF, forward: 5′-AGCCGGTCAAGGCACCTGGG-3′ and reverse 5′-TCAGAAGTGAGGGGAGGCCTGCAGGAAGGGCCTCCAGGTT-3′ real-time PCR primer: FAM83A, forward: 5′-CCCATCTCAGTCACTGGCATT-3′ and reverse: 5′-CCGCCAACATCTCCTTGTTC-3′ ABCG2 forward: 5'-TGGTGTTTCCTTGTGACACTG-3′ and reverse: 5′-TGAGCCTTTGGTTAAGACCG-3′ BMI1 forward: 5′-TCGTTGTTCGATGCATTTCT-3′ and reverse: 5′-CTTTCATTGTCTTTTCCGCC-3′ SOX2 forward: 5′-GCTTAGCCTCGTCGATGAAC-3′ and reverse: 5′-AACCCCAAGATGCACAACTC-3′ OCT4 forward: 5′-GGTTCTCGATACTGGTTCGC-3′ and reverse: 5′-GTGGAGGAAGCTGACAACAA-3; NANOG forward: 5′-ATGGAGGAGGGAAGAGGAGA-3′ and reverse: 5′-GATTTGTGGGCCTGAAGAAA-3′ GAPDH forward: 5′-AATGAAGGGGTCATTGATGG-3′ and reverse: 5′-AAGGTGAAGGTCGGAGTCAA-3′. FAM83A primer used for genomic copy number detection: forward: 5′-CGCCACTGTGTACTTCCAGA-3′ and reverse: 5′-TCCACATCCGTGAACACATC-3′, FAM83A RNAi#1: 5′-GCACAACAACATCAGAGACCT-3′; FAM83A RNAi#2: 5′-GACTGGAGATTTGTCCTGTCT-3′.

### Plasmids, retroviral infection and transfection

The human FAM83A gene was PCR-amplified from cDNA and cloned into pMSCV retroviral vector (Clontech, Mountain View, CA, USA). ShRNAs targeting FAM83A were cloned into the pSuper-retroviral vector. Transfection of plasmids was performed using the Lipofectamine 3000 reagent (Invitrogen, Carlsbad, CA, USA) according to the manufacturer’s instruction. Stable cell lines expressing FAM83A and FAM83A shRNA(s) were generated via retroviral infection as previously described,^45^ and were selected with 0.5 μg/ml puromycin for 10 days.

### Immunohistochemistry

Immunohistochemistry analysis was performed on the 103 paraffin-embedded pancreatic adenocarcinoma tissues, using anti-FAM83A antibody (Sigma). In brief, paraffin-embedded specimens were cut into 4-μm sections and baked at 65 °C for 30 min. The sections were deparaffinized with xylenes and rehydrated. Sections were submerged into EDTA antigenic retrieval buffer and microwaved for antigenic retrieval. The sections were treated with 3% hydrogen peroxide in methanol to quench the endogenous peroxidase activity, followed by incubation with 1% bovine serum albumin to block the nonspecific binding. Rabbit anti- FAM83A (1:500; Sigma) was incubated with the sections overnight at 4 °C. For negative controls, the IgG antibody or normal goat serum was co-incubation at 4 °C overnight preceding the immunohistochemical staining procedure. After washing, the tissue sections were treated with biotinylated anti-rabbit secondary antibody (Zymed, San Francisco, CA, USA), followed by further incubation with streptavidin-horseradish peroxidase complex (Zymed). The tissue sections were immersed in 3-amino-9-ethyl carbazole and counterstained with 10% Mayer's hematoxylin, dehydrated, and mounted in Crystal Mount.

The degree of immunostaining were reviewed and scored separately by two independent pathologists blindly. The scores were determined by combining the proportion of positively stained tumor or normal pancreatic epithelial cells and the intensity of staining. Cell proportions were scored as follows: 0, no positive cells; 1, <10% positive cells; 2, 10–35% positive cells; 3, 35–75% positive cells; 4, >75% positive cells. Staining intensity was graded according to the following standard: 1, no staining; 2, weak staining (light yellow); 3, moderate staining (yellow brown); 4, strong staining (brown). The staining index (SI) was calculated as the product of the staining intensity score and the proportion of positive cells. Using this method of assessment, we evaluated protein expression in benign pancreatic epithelia and malignant lesions by determining the SI, with possible scores of 0, 2, 3, 4, 6, 8, 9, 12 and 16. Then the median value, which SI=8, was chosen as the cut off value. Therefore, samples with a SI⩾8 were determined as high expression and samples with a SI<8 were determined as low expression.

### Sphere formation assay

Five hundred cells were seeded in six-well ultralow cluster plates (Corning, NY, USA) for 10 days. Spheres were cultured in Dulbecco’s modified Eagle’s medium/F12 serum-free medium (Invitrogen, Grand Island, NY, USA) supplemented with 2% B27 (Invitrogen, Grand Island, NY, USA), 20 ng/ml of EGF, and 20 ng/ml of bFGF (PeproTech, Offenbach, Germany), 0.4% bovine serum albumin (Sigma) and 5 μg/ml insulin.

### Chemical reagents

Gemcitabine (Gemzar, Lilly SA, Alcobendas, Spain) and 5-FU (Sigma; 03738) were dissolved in phosphate-buffered saline with concentration of 50 μM. β-Catenin/TCF inhibitor (FH535)(S7484), TGF-β inhibitor (S2704) were purchased from Selleck (Houston, TX, USA).

### Xenografted tumor

The male/female BALB/c nude mice (6–7 weeks of age, 18–20 g) were randomly divided into 15 groups (*n*=6 per group). The indicated cells were inoculated with Matrigel subcutaneously into the inguinal folds of nude mice. Tumor volume was determined using external caliper and calculated using the equation (L × W^2^)/2. The mice were killed 31 days after inoculation, tumors were excised and subjected to pathologic examination. In the experiment testing, the chemoresistance effect of FAM83A, the BALB/c nude mice were implanted subcutaneously with the indicated cells (1 × 10^6^) in order to rapidly induce exponentially growing tumors. When tumors reached a volume of approximate 100 mm^3^, animals were randomly assigned to five groups (*n*=6 per group), followed by intraperitoneal injection of Gemcitabine (80 mg/kg) twice a week. On day 43, animals were killed, and tumors were excised, weighed and subjected to pathological examination. All experimental procedures were conducted in accordance with the Guide for the Care and Use of Laboratory Animals and conformed to our institutional ethical guidelines for animal experiments.

### Flow cytometric analysis

Cells were dissociated with trypsin and re-suspended at 1 × 10^6^ cells/ml in Dulbecco’s modified Eagle’s medium containing 2% fetal bovine serum and then pre-incubated at 37 °C for 30 min with or without 100 μM verapamil (Sigma-Aldrich, Deisenhofen, Germany) to inhibit ABC transporters. The cells were subsequently incubated for 90 min at 37 °C with 5 μg/ml Hoechst 33342 (Sigma-Aldrich). Finally, the cells were incubated on ice for 10 min and washed with ice-cold phosphate-buffered saline before flow cytometry analysis. The data were analyzed by Summit5.2 (Beckman Coulter, Indianapolis, IN, USA).

### Luciferase assay

Ten thousand cells were seeded in triplicate in 48-well plates and allowed to settle for 24 h. One hundred nanograms of luciferase reporter plasmids or the control-luciferase plasmid, plus 5 ng of pRL-TK renilla plasmid (Promega, Madison, WI, USA), were transfected into pancreatic adenocarcinoma cells using the Lipofectamine 3000 reagent (Invitrogen, Carlsbad, CA, USA) according to the manufacturer’s recommendation. Luciferase and renilla signals were measured 48 h after transfection using the Dual Luciferase Reporter Assay Kit (Promega) according to a protocol provided by the manufacturer. Three independent experiments were performed, and the data are presented as mean±s.d.

### Statistical analysis

Statistical tests for data analysis included Fisher’s exact test, log-rank test, chi-square test and Student’s two-tailed *t*-test. Multivariate statistical analysis was performed using a Cox regression model. Statistical analyses were performed using the SPSS 11.0 statistical software package for Windows SPSS Inc. (Chicago, IL, USA). Data represent mean±s.d. *P*<0.05 was considered statistically significant.
