# Supplementary material for: FAM83A is amplified and promotes cancer stem cell-like traits and chemoresistance in pancreatic cancer
Source: Oncogenesis. 2017 Mar 13;6(3):e300–. doi: 10.1038/oncsis.2017.3 (PMC5533946; doi:10.1038/oncsis.2017.3)
Supplement: Supplementary Information [file oncsis20173x1.doc]

**Supplementary Information**

**Supplementary Figure Legends**

**Figure S1. *FAM83A* mRNA is overexpressed in pancreatic cancer.** Real-time PCR analysis of *FAM83A* expression in two primary normal HPDEC lines and ten cultured pancreatic cancer cell lines and in ten primary pancreatic cancer tissues (T) and the matched adjacent non-tumor tissues (ANT). Transcript levels were normalized to *GAPDH*. Error bars represent the mean ± SD of three independent experiments; **P* < 0.05.

**Figure S2. (a–b).** **Real-time PCR analysis of the expression of the pluripotency- associated markers *ABCG2*, *BMI1*, *SOX2*, *OCT4* and *NANOG*.** Error bars represent the mean ± SD of three independent experiments; **P* < 0.05.

**Figure S3. (a)** Heat map of reverse transcription (RT)-PCR analysis of the downstream target genes of the TGF-β or Wnt/β-catenin signaling pathways in the indicated cells compared to the corresponding control cells. Pseudo-color scale values were log2-transformed. **(b)** Kaplan-Meier overall survival curves for patients with pancreatic cancer with low vs. high p-Smad3 expression (*n* = 103; *P* < 0.001, log-rank test; left) and nuclear β-catenin expression (*n* = 103; *P* < 0.001, log-rank test; right). **(c)** Correlations between FAM83A levels and p-Smad3 and nuclear β-catenin expression in pancreatic cancer clinical tissues.

**Figure S4. The *FAM83A* locus is amplified in multiple types of human cancer. (a-b)** Analysis of TCGA datasets indicating *FAM83A* copy number variation (CNV) (a) and *FAM83A* mRNA (b) were aberrantly upregulated in subsets of primary tumors, including LUAD, CESC, BLCA, BRCA, COAD, ESCA, HNSC and UCEC. **(c)** Positive correlation between *FAM83A* CNV and *FAM83A* mRNA expression in LUAD and CESC in the TCGA dataset. **(d)** Kaplan-Meier overall survival (OV) and disease-free survival (DFS) curves for patients with low or high FAM83A expression. **(e)** Relative luciferase activity of TGF-β and TOP/FOP reporter genes in FAM83A-overexpressing or -silenced cells, or control LUAD (A549 and H1299) cells and CESC (HeLa and CaSki) cells. **(f)** Numbers of tumorspheres formed by FAM83A-overexpressing, -silenced cells, and control cells. Each bar represents the mean ± SD of three independent experiments; **P* < 0.05.

**Supplementary Tables**

**Supplementary Table S1**. Clinicopathological characteristics and expression of FAM83A in studied pancreatic adenocarcinoma patients

| **Factor** | **No.** | **(%)** |
| --- | --- | --- |
| **Gender** |  |  |
| Male | 58 | 56.3 |
| Female | 45 | 43.7 |
| **Age (years)** |  |  |
| ≤65 | 43 | 41.7 |
| >65 | 60 | 58.3 |
| **Clinical stage** |  |  |
| I | 12 | 11.7 |
| II | 39 | 37.9 |
| III | 22 | 21.3 |
| IV | 30 | 29.1 |
| **T classification** |  |  |
| T_1_ | 16 | 15.5 |
| T_2_ | 42 | 40.8 |
| T_3_ | 25 | 24.3 |
| T_4_ | 20 | 19.4 |
| **N classification** |  |  |
| N_0_ | 75 | 72.8 |
| N_1_ | 28 | 27.2 |
| **M classification** |  |  |
| No | 67 | 65.0 |
| Yes | 36 | 35.0 |
| **Histological differentiation** |  |  |
| Well | 27 | 26.2 |
| Moderate | 53 | 51.5 |
| Poor | 23 | 22.3 |
| **Vital status** |  |  |
| Alive | 28 | 27.2 |
| Dead | 75 | 72.8 |
| **Expression of FAM83A** |  |  |
| Low expression | 46 | 44.7 |
| High expression | 57 | 55.3 |

**Supplementary Table S2**. Correlation between the clinicopathological features and expression of FAM83A

| **Patient characteristics** | | **FAM83A expression** | | ***P*-value** |
| --- | --- | --- | --- | --- |
|  |  | **Low** | **High** |  |
| **Gender** | Male | 24 | 34 | 0.549 |
|  | Female | 22 | 23 |  |
| **Age (years)** | ≤65 | 21 | 22 | 0.548 |
|  | >65 | 25 | 35 |  |
| **Clinical stage** | I | 9  132  62 | 3  44 | 0.005 |
|  | II | 22 | 17 |  |
|  | III | 8 | 14 |  |
|  | IV | 7 | 23 |  |
| **T classification** | T_1_ | 11  64  106 | 5  18 | 0.010 |
|  | T_2_ | 23 | 19 |  |
|  | T_3_ | 7 | 18 |  |
|  | T_4_ | 5 | 15 |  |
| **N classification** | N_0_ | 40  204 | 35  0 | 0.004 |
|  | N_1_ | 6 | 22 |  |
| **M classification** | No | 36 | 31 | 0.013 |
|  | Yes | 10 | 26 |  |
| **Histological**  **differentiation** | Well | 17  174 | 10 | 0.014 |
|  | Moderate | 24 | 29 |  |
|  | Poor | 5 | 18 |  |
| **Vital status** | Alive | 21 | 7 | < 0.001 |
|  | Dead | 25 | 50 |  |

**Supplementary Table S3**. Univariate and multivariate analysis of different prognostic parameters associated with overall survival in patients with pancreatic adenocarcinoma by Cox-regression analysis

|  | **Univariate analysis** | | **Multivariate analysis** | |
| --- | --- | --- | --- | --- |
|  | ***P*** | **Hazard ratio**  **(95% CI)** | ***P*** | **Hazard ratio**  **(95% CI)** |
| **Clinical stage** | 0.001 | 2.194  (1.571-3.553) | 0.019 | 1.528  (0.531-2.721) |
| I |  |  |  |  |
| II |  |  |  |  |
| III |  |  |  |  |
| IV |  |  |  |  |
| **T classification** | 0.015 | 1.831  (0.635-2.922) | 0.048 | 0.682  (0.388-0.954) |
| T_1_ |  |  |  |  |
| T_2_ |  |  |  |  |
| T_3_ |  |  |  |  |
| T_4_ |  |  |  |  |
| **N classification** | < 0.001 | 2.931  (2.223-4.012) | 0.004 | 2.085  (1.405-3.186) |
| No |  |  |  |  |
| N1 |  |  |  |  |
| **M classification** | < 0.001 | 3.52  (2.219-5.162) | <0.001 | 2.352  (1.820-3.380) |
| M0 |  |  |  |  |
| M1 |  |  |  |  |
| **FAM83A expression** | < 0.001 | 2.836  (1.876-4.286) | 0.002 | 2.212  (1.420-3.411) |
| Low expression |  |  |  |  |
| High expression |  |  |  |  |

**Supplementary Table S4**. Univariate and multivariate analysis of different prognostic parameters associated with disease-free survival in patients with pancreatic adenocarcinoma by Cox-regression analysis

|  | **Univariate analysis** | | **Multivariate analysis** | |
| --- | --- | --- | --- | --- |
|  | ***P*** | **Hazard ratio**  **(95% CI)** | ***P*** | **Hazard ratio**  **(95% CI)** |
| **Clinical stage** | 0.009 | 2.032  (1.398-3.276) | 0.024 | 1.412  (0.503-2.598) |
| I |  |  |  |  |
| II |  |  |  |  |
| III |  |  |  |  |
| IV |  |  |  |  |
| **T classification** | 0.023 | 1.681  (0.712-2.421) | 0.036 | 0.767  (0.471-1.163) |
| T_1_ |  |  |  |  |
| T_2_ |  |  |  |  |
| T_3_ |  |  |  |  |
| T_4_ |  |  |  |  |
| **N classification** | < 0.001 | 2.521  (2.021-4.278) | 0.006 | 2.287  (1.635-3.365) |
| No |  |  |  |  |
| N1 |  |  |  |  |
| **M classification** | < 0.001 | 2.671  (2.209-4.541) | 0.001 | 2.189  (1.654-3.187) |
| M0 |  |  |  |  |
| M1 |  |  |  |  |
| **FAM83A expression** | < 0.001 | 3.267  (2.165-5.145) | 0.010 | 2.412  (1.582-3.874) |
| Low expression |  |  |  |  |
| High expression |  |  |  |  |
