# Supplementary figures and images for: FAM83A is amplified and promotes cancer stem cell-like traits and chemoresistance in pancreatic cancer
Source: Oncogenesis. 2017 Mar 13;6(3):e300–. doi: 10.1038/oncsis.2017.3 (PMC5533946; doi:10.1038/oncsis.2017.3)

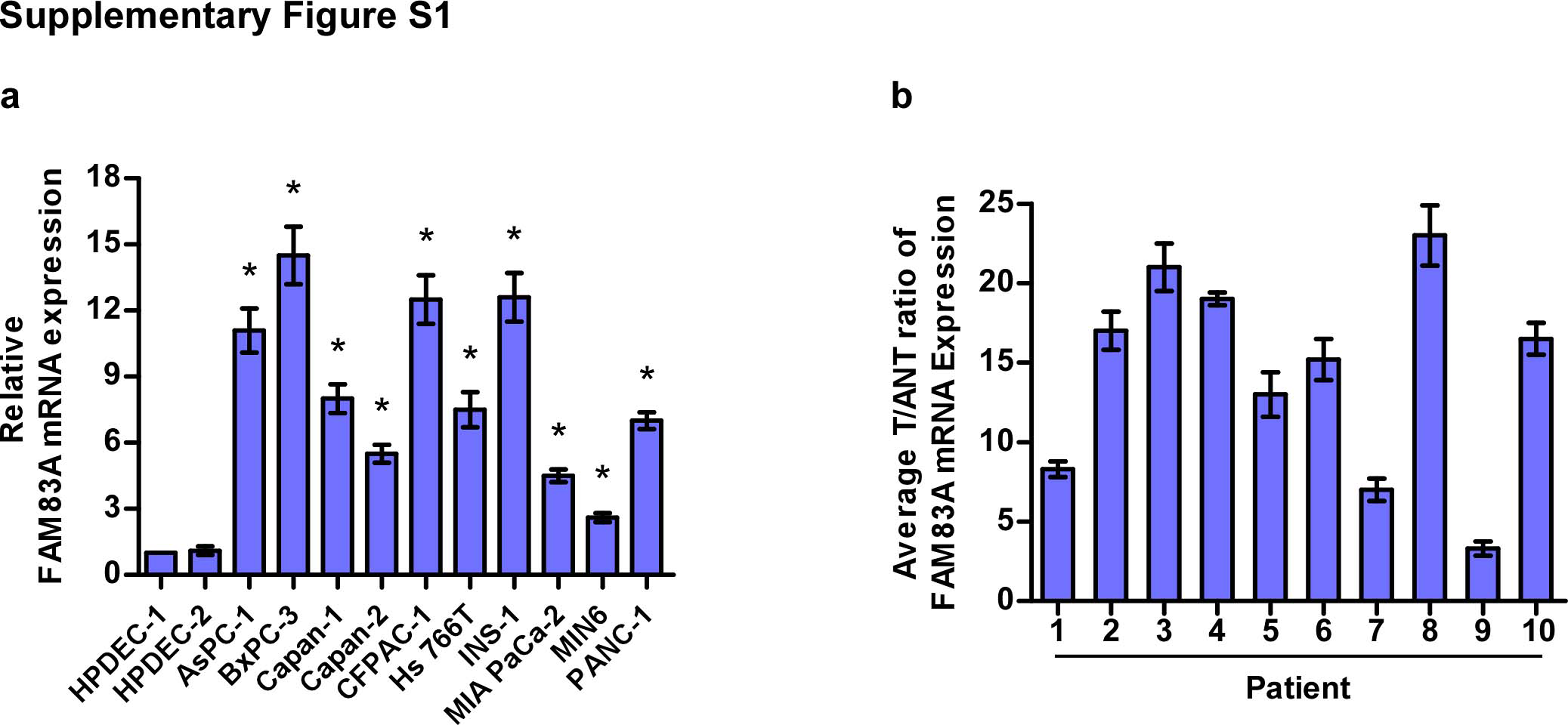

Supplement: Supplementary Figure 1 [file oncsis20173x2.tif]
